# The population of high CTLA‐4 expressing with CD56 positive T cell is diminished in Alzheimer’s disease patients

**DOI:** 10.1002/alz.086100

**Published:** 2025-01-03

**Authors:** Cheng‐I Chu, Ching‐Tse Wu, Feng‐ Yu Wang, Hui‐ Yu Yang, Chien‐Chung Chang, Chuang‐Rung Chang

**Affiliations:** ^1^ National Taiwan University Hospital Hsinchu Branch, Hsinchu Taiwan; ^2^ National Tsing Hua University, Hsinchu Taiwan; ^3^ National Taiwan University, Taipei Taiwan

## Abstract

**Background:**

Abnormal brain inflammation is an important feature of Alzheimer’s disease (AD). Central nervous system (CNS) inflammation is highly related to immune cell activation. Homeostasis of immune cell activity regulation is crucial for CNS autoimmune response. Our previous study found that the expression of immune checkpoint PD‐L1/PD‐1 on T cells changed along with different stages of AD patients. We assayed the other immune checkpoint molecule, CTLA‐4, and its functional characteristics to characterize the changes of T cell populations of peripheral blood in AD patients.

**Method:**

AD patients (n = 14) and healthy elderly (n = 16) were enrolled. White blood cells collecting from blood were labelled with fluorescent antibodies and assayed by flow cytometry. To characterize changes in specific T‐cell populations and features, we used t‐distributed stochastic neighbor embedding (tSNE). The analysis results of all volunteers were pooled, aggregated, and integrated with different phenotypic and functional characteristics. Distribution patterns were delineated between AD and healthy elderly, differences were compared, and molecular features were identified for further subpopulation analysis.

**Result:**

(A) In the phenotypic and functional characteristics feature distribution of T cells, the subpopulation distribution difference between AD and healthy elderly was related to the expression intensity of NCAM (Neural cell adhesion molecule, CD56). (B) Further distinguishing the T cell population with positive expression of CD56, three subpopulations were found, subpopulation A, B and C. Subpopulation A decreased in AD patients, subpopulation C increased in AD patients, and subpopulation B had no obvious difference. Prominent difference of these three subpopulations was the expression level of CTLA‐4. Subpopulation A was CTLA‐4^High^CD56+ T cell subset; subpopulation B was CTLA‐4^Dim^CD56+ T cell subset; subpopulation C was CTLA‐4^Medium^CD56+ T cell subset.

**Conclusion:**

CD56 (NCAM) positive T cells with high expression of CTLA‐4 decreased in AD patients. CTLA‐4 played important role in T cell priming brake, and competed B7 ligand with CD28 to suppress T cell activation. Decrease of CTLA‐4 expression on CD56+ T cells indirectly promote the immune activation. Our research indicated immune imbalance in peripheral blood of AD patients. Changes of T cell subpopulations may be biomarkers of AD, and maintaining homeostasis of immunity may be potential treatment of AD in future.

